# Toxic Leukoencephalopathy due to Suspected Levamisole-adulterated Cocaine

**DOI:** 10.1007/s00062-023-01358-z

**Published:** 2023-11-14

**Authors:** Rafael Willems, Se-Jong You, Friederike Vollmer, Elke Hattingen, Stefan Weidauer

**Affiliations:** 1 Institute of Neuroradiology, University Hospital Frankfurt, Goethe University, Frankfurt am Main, Germany; 2 Clinic of Neurology, University Hospital Frankfurt, Goethe University, Frankfurt am Main, Germany

## Introduction

Cocaine is known to cause several neurological complications including ischemia, hemorrhage, vasculitis, and toxic leukoencephalopathy. The leukoencephalopathy is often suspected to be triggered by levamisole, an increasingly used diluent. Levamisole, developed in the 1960s originally for its immunomodulatory effects, has been used in the treatment of rheumatoid arthritis, nephritic syndrome and as a chemotherapeutic agent, for example [1]; however, it may cause complications, e.g., neutropenia, thrombocytopenia, cutaneous complications or arthralgia [2]. Moreover, due to its therapeutic use, levamisole is known to trigger multifocal inflammatory leukoencephalopathy probably due to increased pathologic immune reactions depending on the hereditary predisposition [3–6]. In 2011, Lynch et al. [7] reported on 203 users with toxic side effects due to levamisole-adulterated cocaine. Because of the short traceability of levamisole showing a half-life period of 5.6 h, the substance could be detected only in approximately a quarter of patients, i.e., 57 out of 203 cases; however, none of them suffered from toxic leukoencephalopathy [2, 7]. To our knowledge, there are only two reported cases that additionally tested positive for levamisole in an emergency setting and one about 2 months after symptom onset due to hair analysis. Nevertheless, it is suspected that the number of leukoencephalopathies caused by the consumption of levamisole-contaminated cocaine is clearly underestimated [8–10]. We add a case of a 29-year-old woman suffering from severe toxic leukoencephalopathy positive for cocaine, while levamisole testing was negative.

## Case Report

The patient suffered from progressive dizziness, behavioral changes, and gait ataxia within 1 day. On admission, she was somnolent, partially disorientated and showed slowed mental state. Cranial nerves, motor function of the limbs, deep tendon reflexes and sensory examination were unremarkable. Severe gait ataxia resulted in inability to walk. Within the next 24 h, there was severe deterioration with coma, deep tendon reflexes of the limbs were exaggerated, and plantar extensor response was bilateral. Extensive laboratory investigations including cerebrospinal fluid (CSF) analysis and microbiological testing were unremarkable. Whereas urine drug screening in an emergency setting on admission was positive for cocaine, additional testing for levamisole was negative. On sequential electroencephalography hypersynchronous epileptic activity as well as nonconvulsive seizures were excluded.

**Fig. 1:**
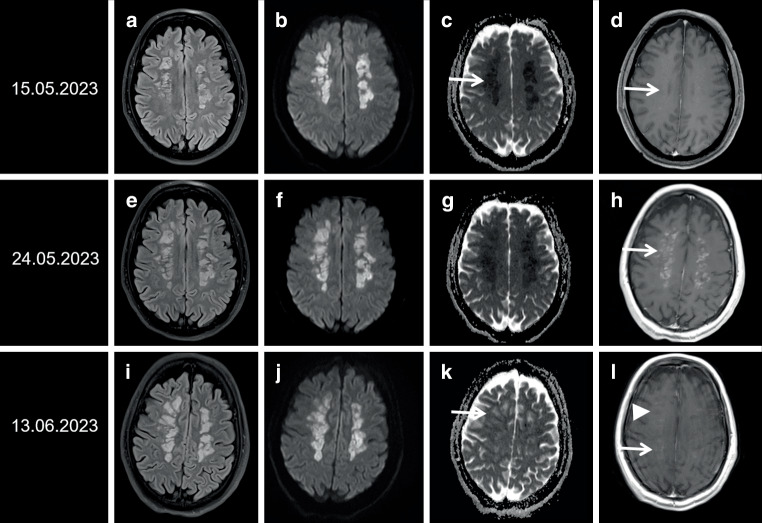
Sequential MR imaging in a 29-year-old woman suffering from severe toxic leukoencephalopathy. **a**, **e**, **i** Axial fluid attenuated inversion recovery (FLAIR); **b**, **f**, **j** and **c**, **g**, **k** axial diffusion weighted images (DWI; b = 1000 s/mm^2^) and corresponding apparent diffusion coefficient (ADC) maps; **d**, **h**, **l** axial T1 weighted images (WI) after administration of contrast agent. **a**–**d** Initial MR imaging 3 days after drug intake showing partially conflating bilateral hyperintense signal changes in the centrum semiovale (**a**) with severe restricted diffusion (**b**, **c**; ADC mean: 0.23 × 10^−3^ mm^2^/s, *arrow*; normal: 0.72 × 10^−3^ mm^2^/s); slight punctuate contrast enhancement (**d**, *arrow*). **e–h** Follow-up imaging on day 12 disclosed progressive hyperintense lesions (**e**, **f**), slight improvement of lowered ADC values (**g**; ADC mean: 0.37 × 10^−3^ mm^2^/s) and distinct patchy contrast enhancement (**h**, *arrow*). **i–l** On day 32 FLAIR images exhibit explicit bilateral hyperintense signal changes (**i**) with “shine through effect” on DWI (**j**), pseudonormalization of ADC values (**k**, *arrow*; range: 0.79–1.44 × 10^−3^ mm^2^/s) and decreasing contrast enhancement (**l**, *arrow*); note inhomogeneous lowering of T1 signal in the centrum semi-ovale (**l**, *arrowhead*)

Whereas initial cranial computed tomography (CT) showed suspected slight confluent hypodense lesions in the supratentorial deep white matter, magnetic resonance imaging (MRI) 24 h later revealed extensive nearly symmetric hyperintense signal changes on T2-weighted images going along with restricted diffusion indicative for toxic leukoencephalopathy (Fig. 1a–d).

Despite treatment with intravenous (i.v.) high dosage corticosteroids, i.e., 1000 mg/day for 7 days, there was no neurological improvement. Furthermore, the patient suffered from several vegetative crises with severe arterial hypertension and tachycardia up to 180 beats per minute. Follow-up MRI 9 days later disclosed progressive voluminous signal alterations in the supratentorial deep white matter on T2-weighted images (WI) sparing the infratentorial brain parenchyma (Fig. 1e–h).

An additional second course of i.v. high-dosage corticoid treatment, i.e., 2000 mg/day for 5 days also failed.

On follow-up 28 days later, neurological examination showed slight recovery of vigilance accompanied by a minimally conscious state. The woman suffered from severe dysarthria, dysphagia, spastic tetraparesis and was transferred to a rehabilitation center. In line with unfavorable neurological outcome, neuroimaging disclosed damaged deep white matter with regression of temporary blood-brain barrier disruption (Fig. 1i–l).

## Discussion

The differential diagnosis of rapidly progressive altered conscious state and gait ataxia comprises different etiologies including vascular and inflammatory diseases, epileptic seizures, metabolic disorders, cardiovascular failure, drug abuse and intoxication [11].

Typical neurological complications due to cocaine abuse are intracerebral and subarachnoid bleeding and ischemia due to hypertensive crises or vasculitis, especially in the watershed areas, the mesencephalon, and the basal ganglia [12, 13]; however, the etiology of cocaine-associated leukoencephalopathy is still unclear. Several authors [13–16] assumed that toxic leukoencephalopathies are not caused mainly by cocaine itself but related to excipients and impurities. Levamisole, a widespread adulterant, which has been significantly linked to leukoencephalopathies as a previous therapeutic drug, seems to be a key player and is probably severely underestimated [3, 9, 17]. Originally developed because of its immunomodulatory effects and formerly used as a chemotherapeutic drug for example, levamisole is still used today as a veterinary anthelminthic drug and illegally as an adulterant in cocaine [1]. Whereas a review of the literature yielded no documented values of contaminated cocaine in Germany, in Luxembourg in 2010 even up to 86% of cocaine samples contaminated with levamisole were reported [18]. The exact mechanism of levamisole in demyelination is not completely understood but is suggested to be due to an increase of a pathologic immune reaction to an unknown antigen in genetically predisposed people rather than to a direct toxic effect [5, 6].

In 2021, a study [19] investigated white matter hyperintensities in 35 cocaine users and 34 healthy controls. While cocaine users showed more white matter hyperintensities in terms of volume, but not in terms of number, users of levamisole-exposed cocaine showed even more affected tissue both in terms of volume and in terms of number.

Although in the presented case urine testing was positive for cocaine but not for levamisole, the suspected etiology of severe neurological deterioration is levamisole-induced toxic leukoencephalopathy due to impressive findings in MRI. While cocaine-induced leukoencephalopathies usually show no contrast enhancement, intermittently there was distinct patchy contrast enhancement on day 12 after the estimated drug intake in the presented case (see Fig. 1). This imaging feature has been previously described as typical of levamisole-induced toxic leukoencephalopathy due to breakdown of the brain-blood barrier [10, 20, 21]. The negative testing for levamisole and its metabolites is most likely due to the short half-life period of levamisole of 5.6 h [2]. Levamisole is detectable in urine up to about 39 h after ingestion, while aminorex as one of its metabolites is detectable in urine up to approximately 54 h after ingestion [22]. Furthermore, detection in plasma is possible up to 36 h for levamisole and up to 30 h for aminorex [23]. Although in the reported patient testing for levamisole was negative, toxic leukoencephalopathy due to this excipient in cocaine users is a likely consequence. Although urine testing for cocaine, levamisole and its metabolites was performed on admission, medical history suggested intake of unpurified cocaine at least 2 days before hospital admission.

Neuroradiological findings in levamisole-induced leukoencephalopathy initially showed extended supratentorial hyperintense signal changes on T2 WI in the deep white matter accompanied by severely restricted diffusion and consecutive lowered apparent diffusion coefficient (ADC) values. Follow-up imaging 9 days later demonstrated disruption of the blood-brain barrier with patchy inhomogeneous contrast enhancement of the lesions. In the subacute stage diffusion weighted imaging (DWI) disclosed pseudonormalization going along with persisting hyperintense signal changes on T2 WI and partially regressive contrast enhancement on T1 WI indicating irreversible tissue damage. In addition, slight bilateral volume loss especially of the caudate nucleus and the corpus striatum were present (not shown) [8, 9]. Similar imaging findings have been reported in carbon monoxide intoxication and heroine abuse, especially “chasing the dragon”, i.e., inhalation of vaporized heroine [24–26]. Further differential diagnoses in acute leukoencephalopathy encompass chemotherapy-associated side effects, posterior encephalopathy, osmotic demyelination, hypoglycemia, hepatic failure, and hypoxic damage [27–32].

## Conclusion

The differential diagnosis of acute onset of impairment of consciousness, progressive gait ataxia and behavioral changes should include levamisole-induced toxic leukoencephalopathy especially in drug addicts. The rate of levamisole-contaminated cocaine in Europe is estimated to be partially as high as 86%; however, routine drug screening in an emergency setting often does not include analysis for levamisole. Furthermore, the half-life period of the substance is very short and an underestimated incidence of levamisole-induced encephalopathy is a likely consequence. Large confluent lesions in the deep white matter with restricted diffusion and disturbance of blood-brain-barrier on MRI are possible imaging findings indicating an unfavorable outcome.
